# Breeding Biology and Variable Mating System of a Population of Introduced Dunnocks (*Prunella modularis*) in New Zealand

**DOI:** 10.1371/journal.pone.0069329

**Published:** 2013-07-09

**Authors:** Eduardo S. A. Santos, Shinichi Nakagawa

**Affiliations:** 1 Department of Zoology, University of Otago, Dunedin, New Zealand; 2 Departamento de Ecologia, Universidade de São Paulo, São Paulo, Brazil; Hungarian Academy of Sciences, Hungary

## Abstract

Species with variable mating systems provide a unique opportunity to investigate whether females receive direct fitness benefits from additional male partners. The direct benefits provide an obvious explanation for why females would breed polyandrously, in a situation where males clearly do not attain their optimal reproductive success. Evidence for these direct benefits is, however, mixed. Here, we present a detailed study of the breeding biology of the dunnock, *Prunella modularis*, which inform an investigation into the effects of the social mating system on the reproductive success in a population of dunnocks in Southern New Zealand. We studied 80 different social groups over the course of three breeding seasons. Dunnocks in our population presented a variable mating system, with socially monogamous (45%), socially polyandrous (54%) and socially polygynandrous (1%) groups being observed in the same breeding season. We did not observe any polygynous social units in our study period although polygyny exists in the population. We found little difference in the numbers of eggs laid, and egg volume between monogamous and polyandrous nests. However, polyandrous groups had better hatching and fledging success than monogamous groups (composite *d* = 0.385, 95% CI: 0.307 to 0.463). Overall our results support the notion that polyandry is beneficial for females.

## Introduction

Usually, there is little intra-specific variation in social breeding systems, with one form (e.g., monogamy) being commonly the only one observed in a population. This is especially the case for bird species, although, in some instances, the genetic breeding system could provide another dimension of variation [1], [2]. When individuals engage in multiple copulations, be it with different social partners or with extra-pair mates, it is thought that they seek additional fitness benefits. While it is clear that males benefit directly from breeding outside of their pairs (i.e. by increasing the number of offspring produced), the search for female fitness benefits of multiple paternity has been more challenging [3]–[5]. In many species, females do not receive obvious direct benefits (e.g., increases in reproductive success of the individual or its offspring), and thus, it is predicted that they should receive indirect genetic benefits [3].

Nevertheless, some species exhibit variable breeding systems, in which two or more types of social units occur during the reproductive season (e.g., pukeko, *Porphyrio porphyrio*, [6]). In these species, the outcome of sexual conflict over mating optima is more readily observed. For instance, some females might breed in socially polyandrous groups, gaining direct fitness benefits conferred by additional paternal care. Other females, in contrast, might be constrained to breeding with a single partner in monogamy. However, studies that investigated the direct benefits of additional male help in species with variable mating system have provided mixed findings. There is little evidence that the additional help provided in polyandrous groups of the ground tit, *Pseudopodoces humilis* [7], and of the moustached warbler, *Acrocephalus melanopogon* [8] is converted into fitness benefits for these polyandrous females. On the other hand, in another species with a variable mating system, the dunnock, *Prunella modularis*, socially polyandrous females fledge more offspring than socially monogamous ones [9]. More investigations are clearly needed to gain better understanding of what benefits, if any, females in variable mating system species gain from breeding polyandrously. Especially, a replication using the same species in a different population is likely to provide us with fresh insights.

In this study, we describe the breeding ecology of dunnocks in one population of these introduced birds in southern New Zealand (NZ). Using this information, we then investigate whether the type of social mating system affects breeding parameters (e.g., clutch size, egg volume, territory size), and hatching and fledging success of dunnocks. Our study provides important information for this species in a non-native range. Moreover, we have a unique opportunity to investigate the fitness benefits of polyandry for a species that has already been extensively studied.

## Methods

### Ethics statement

All the research reported here was conducted with the approval of the Animal Ethics Committee of the University of Otago (permit no. 08/09) and birds were banded under the New Zealand National Bird Banding Scheme Institutional Permit to Band Birds no. 2008/075. We thank the Dunedin Botanic Garden and its staff for allowing us to conduct this research on their grounds.

### Study species

In-depth studies of dunnocks have been conducted in its native range in Europe (e.g., [10]–[16]). These studies have identified that dunnocks present a variable mating system within populations. Dunnocks form breeding groups that are composed of unrelated adults and can range from monogamy, polyandry (one female associated with two or three males concurrently), polygyny (one male associated with two or more females) and polygynandry (two or more females paired with two or more males) (see [10], [17], [18]). Although dunnocks have been well studied in Europe and considered to be one of the classic model species in the study of behavioural ecology, no study of its breeding ecology and behavioural ecology has been conducted in its introduced range in New Zealand (but see [8] for a study of the dunnock's diet). Thus, little is known of the effects that the release of the species in a new environment could have had on its breeding ecology and behaviour. A better understanding of the dunnock's breeding biology in New Zealand would provide important information for future studies of its behaviour from a comparative point of view with the previous European studies.

The dunnock is abundant in New Zealand, due to their deliberate introduction by English settlers in the 19th century [19]–[21], with approximately 640 dunnocks released at five different locations during this period [19], [20], [22]. Data from a dunnock population in Southern New Zealand suggest little genetic and morphological divergence from a native population in England [23].

### Study area

This study was conducted at the Dunedin Botanic Garden (45.87° S, 170.50° E), Dunedin, New Zealand. We focused on a 7.1 ha area of the Botanic Garden that contained a diversity of human-made habitats, including open lawns, hedgerows, flowerbeds, and open woodland. The study was conducted between July 2009 and January 2012, comprising three breeding seasons: 2009–2010, 2010–2011 and 2011–2012.

### Nest monitoring and general field methods

In order to obtain breeding ecology data, we searched for dunnock nests during spring and summer, from September to January, either by walking through the study area and checking the vegetation for potential nests, or by following adults carrying nesting materials. We usually located nests before egg laying, as we were able to observe nest-building adults. Once we found a nest, we recorded its height above the ground, and for a subset of nests we measured four structural variables (inside cup diameter and cup depth, and outside cup diameter and cup height; ±0.01 cm). We used these data to describe the general strata of the vegetation that dunnocks use for nesting, and also the basic structural characteristics of the nests.

To collect breeding parameter data we monitored nests as follows. From the moment we found a nest, we checked it every two days until the first egg was laid (we considered nests to be active when we found either eggs or nestlings in them). During the egg laying stage, we checked nests daily in order to record the laying order. We measured each egg with dial callipers (length and width; ±0.1 mm), weighed on an electronic scale (±0.01 g), and sequentially numbered with a permanent felt-tip marker to indicate the position in the laying order. We used the length and width of eggs to estimate their volume (mm^3^) based on Hoyt's formula: 0.51× length × width^2^ [24]. During the incubation period, we visited nests every two days until the first egg hatched. Most eggs in a nest hatched on the same day, and in most cases we were not able to match nestlings to the eggs. We also recorded the fate of each egg (disappeared, unhatched, hatched) and nestling (died in nest, disappeared, fledged). We marked all nestlings at hatching by uniquely clipping their superciliary feathers [25] and by applying a unique combination of dots with a permanent felt-tip marker to their nape for individual recognition. Before nestlings fledged, we colour banded them at nine days of age (age at hatching: zero day old) with a combination of three colour and one numbered aluminium bands.

### Colour banding and mating system assessment

We captured adult dunnocks throughout our study site during both the non-breeding season and the breeding season by placing mist nets at frequently used flight paths (i.e. usually at territory borders and around nesting sites). We banded each dunnock with a unique combination of three colour and one numbered aluminium bands for individual recognition.

We characterized the social mating system of each breeding group by visiting nests at least five times during the nest cycle and recording the number of unique adults present in the territory that interacted with each other in social behaviours (e.g., foraging jointly, joint territory defence, mating, parental care). Moreover, to ascertain the social mating system, we also used the identity of adults that provided parental care to nestlings (i.e. through video recordings of parental behaviour). We did not characterize the genetic mating system of the groups in this study although we plan to do so in a future study.

We recorded GPS relocations of breeding colour-banded adults in order to estimate territory sizes. We estimated territory size per group using the minimum convex polygon method [26] implemented in the *adehabitatHR* package [27] in *R* (version 2.13.0; [28]). In our estimates of territory size, we used the locations where we saw all birds from a group engaging in the following behaviours: singing, fighting, patrolling, and/or foraging.

We estimated the density of breeding males using the number of males assigned to a breeding territory during each breeding season (when males had multiple breeding attempts, they were included only once in the estimate). We present the average density of breeding males per hectare over the three breeding seasons. Furthermore, we estimated the adult sex-ratio of the population as the proportion of breeding unique males to unique females in each breeding season.

### Statistical analyses

To investigate the effect of the social mating system on the different breeding parameters, we used Bayesian mixed-effect models (BMM) implemented using the *MCMCglmm* package [29] in *R* (version 2.13.0; [28]). We built BMMs for the following breeding parameters as response variables: territory size (hectares; Gaussian link function), laying date of nests (Julian date; Gaussian link function), number of eggs laid (clutch size; Poisson link function), egg volume (mm^3^; Gaussian link function), proportion of eggs that hatched (Binomial link function), and proportion of chicks that fledged (Binomial link function). We fitted all BMMs with the social mating system as a categorical fixed effect with two levels (polyandrous and monogamous; we did not include polygynandrous mating system in this analysis due to low sample size [one group and four nesting attempts]). We also included the breeding season in which the data was collected as a categorical fixed effect with three levels in all models (Season A for nests found in 2009–2010, Season B for nests found in 2010–2011 and Season C for nests found in 2011–2012). We removed the breeding season fixed effect from final reported models if its effect was non-significant for a given response variable (in all occasions where it was non-significant, the Deviance Information Criterion (DIC) supported the most parsimonious model) [30]. In the BMMs, we included the breeding group identity as a random effect to account for the lack of independence between measures. Moreover, we inspected plots of the models' residuals for heteroscedasticity [31]. When there was evidence for heteroscedasticity in the data (e.g., the variance in territory size of polyandrous groups was greater than that of monogamous groups), we accounted for it by modelling the residual variance structure to allow the variance in the effects to be different for each level of the fixed effect. We used a sample of 1,000 iterations from which to estimate the BMM model estimates. In order to obtain this sample for each model, we used the following range of model parameters: number of iterations  = 1,000,000–2,000,000; burn-in  = 900,000–1,000,000; and thinning of 100 or 1,000. We used an Inverse Gamma prior for all models.

To estimate nest daily survival rates (DSR), we used the package *RMark* (version 2.06; [32] for *R* (version 2.13.0; [28]). We used the method developed by [33] to evaluate variation in DSR as a function of the type of social mating system that nests belonged to. We used Pearson's correlation to evaluate the magnitude of the association between nest structural characteristics. All descriptive statistics are presented as means (standard deviations) and sample size, unless specified otherwise. Model estimates (slopes of the differences between factor levels) are presented as posterior means (standard deviations) and 95% credible intervals (95% CI). We considered parameters with 95% CIs not spanning zero to be statistically significant. For each result investigating the effect of social mating system on a breeding parameter, we present a standardized effect size statistic. We use Cohen's *d* as the effect size metric in order to present results in an ‘effective’ thinking framework [34]. Cohen proposed that estimates of *d* of 0.2, 0.5 and 0.8 are considered ‘small’, ‘medium’ and ‘large’, respectively [35]. We use these values as benchmarks in order to assess the magnitude of our findings. Finally, we use a within-study meta-analysis approach [36] to calculate a composite Cohen *d* value for hatching and fledging success, which is a measure of the overall effect of social mating system on reproductive success.

## Results

### Breeding season phenology and nest characteristics

**Figure 1:**
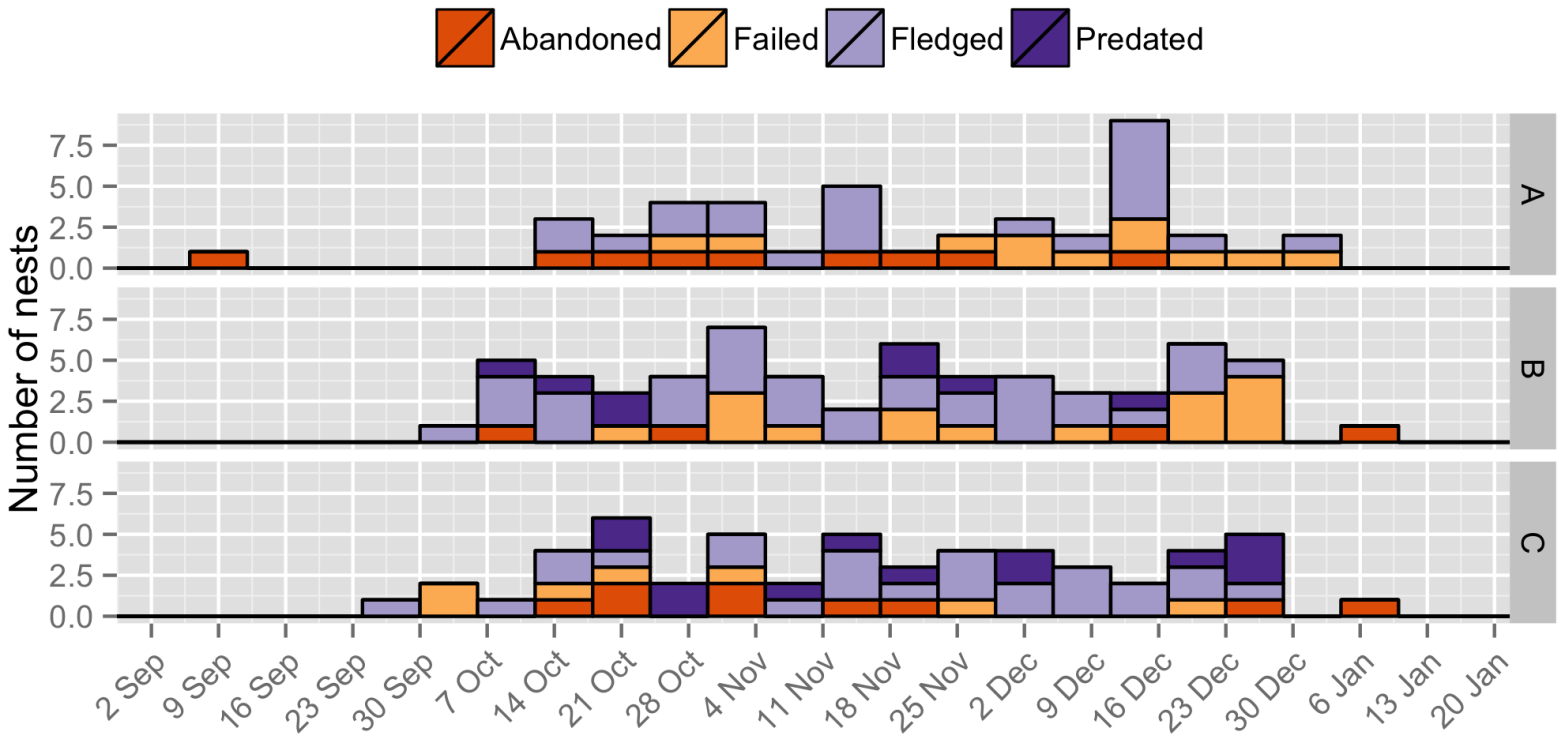
The timing of breeding in dunnocks. The timing of breeding by a population of dunnocks in Dunedin, New Zealand during the breeding seasons of (a) 2009–2010, (b) 2010–2011, and (c) 2011–2012. Bars summarise the number of active nests per week, based on the date that the first egg was laid. When the date of the first egg was unknown, it was estimated by backtracking from the average length of the incubation or nestling periods. For nests that never hatched, we used the date in which the nest was encountered as a proxy for the first egg date. The different fillings of the bars represent the outcome of the nests (i.e., abandoned, failed [e.g. unfertilized eggs, dead nestlings due to starvation or extreme weather], fledged, or predated).

We found and monitored 42 active nests in 2009–2010, 62 in 2010–2011 and 54 in 2011–2012 (henceforth, Season A, Season B and Season C, respectively; Figure 1). Breeding groups began to build nests in the first week of October in Season A, the third week of September in Season B, and again in the third week of September in Season C. Behavioural observations of colour-banded individuals during the nest-building stage revealed that only females built nests. These observations also showed that females built nests quickly (mean number of days to build nest: Season A: 7.8 (2.77) days, *n* = 5; Season B: 5.5 (1.71) days, *n* = 10; Season C: 5.6 (1.50) days, *n* = 11). The period of egg laying extended for 86 days in Season A (from the second week of October 2009 to the first week of January 2010), 101 days in Season B (from the last week of September 2010 to the second week of January 2011) and 88 days in Season C (from the last week of September 2011 to the last week of December 2011). Groups frequently re-nested in the same breeding season after depredation of the nest (up to three times), and also after successfully fledging young. Fifty percent (21/42) of the nesting territories of Season A were re-used in Season B, and 46.7% of those (29/62) from Season B were reused in Season C; all re-nesting events (both within- and between seasons) by the same group took place in immediately adjacent nests to the old ones (i.e., no nests were re-used).

Nests were open-cup structures of sticks, lined mainly with mosses and feathers. Nest height ranged from 0.16 to 3 m above the ground (mean height: 1.21 (0.67) m, *n* = 100). We measured a subset of nest for their structural dimensions (*n* = 10): inside cup diameter (mean: 3.75 (0.40) cm); inside cup depth (mean: 6.55 (0.93) cm); outside cup diameter (mean: 12.63 (1.58) cm); outside cup height (mean: 7.52 (1.22) cm). All the nest dimensions were positively associated (0.34<*r*<0.74).

### Social mating system, territories, and adult sex-ratio

We captured and colour banded 126 adults and 202 nestlings during all three breeding seasons. We confidently assessed the social mating system of 85% of the active nests (134/158), as colour-banded individuals attended them. A total of 80 unique breeding groups attended this subset of nests. We observed the following frequency of social mating systems in our population: 45.0% (36/80) of the breeding groups were monogamous pairs, 53.7% (43/80) were polyandrous groups composed of one female and two or three males, and 1.3% (1/80) was a polygynandrous group composed of two females and two males. We did not observe any polygynous breeding group. Group composition persisted throughout the year (i.e. we did not observe any individuals switching groups outside of the breeding season).

**Figure 2:**
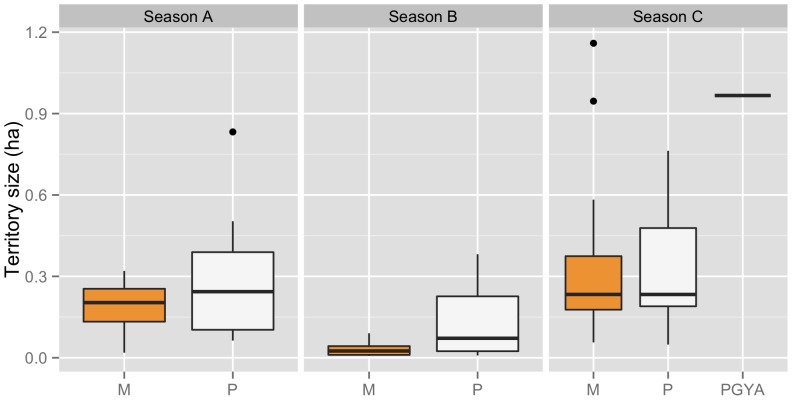
Territory size in dunnocks by type of mating system. Territory size in hectares of monogamous (M), polyandrous (P), and polygynandrous (PGYA) social groups of dunnocks in Dunedin, New Zealand during the 2009–2010 (Season A), 2010–2011 (Season B) and 2011–2012 (Season C) breeding seasons.

Dunnocks in our population are year-round residents and defend territories throughout the year. Territories encompassed lawns, flowerbeds, and a variety of gardens containing shrubs, herbs and trees. For most groups, the territory borders made contact with at least another territory, indicating that all suitable nesting habitat was occupied. The effect size of mating system on territory size was ‘small’ (*d* = 0.008) and the difference was not statistically significant between polyandrous groups and monogamous groups (Table 1; Figure 2). There was, however, a significant negative effect of the breeding season on territory size, with territories in Season B being smaller than those of both Season A and Season C for both polyandrous and monogamous groups (Gaussian BMM: β_breeding season B_: −0.202 (0.052), 95% CI: −0.302 to −0.097; β_breeding season C_: 0.078 (0.046), 95% CI: −0.002 to 0.176; Figure 2). Over the course of both seasons, our study site had an average density of 5.047 (0.861) breeding males per hectare, and the adult breeding population was male-biased (average proportion of adult males, ASR = 0.569 (0.009)).

**Table 1:** Breeding parameters by the type of social mating system in dunnocks.

	Polyandrous	Monogamous	Effect size (β (SD))	95% CI	Cohen's d (SE), 95% CI
Territory size (ha)	0.246 (0.216), n = 32	0.244 (0.258), n = 30	0.050 (0.040)	−0.029 to 0.127	0.008 (0.254), −0.489 to 0.506
Laying date (days since the beginning of the breeding season)	44.375 (25.13), n = 40	38.43 (26.221), n = 41	5.286 (5.916)	−6.083 to 16.804	0.229 (0.222), −0.207 to 0.666
Clutch size (eggs)	3.576 (0.648), n = 61	3.507 (0.841), n = 69	0.029 (0.092)	−0.152 to 0.205	0.090 (0.175), −0.253 to 0.435
Egg volume (mm^3^)	2076 (165), n = 198	2062 (169), n = 229	9.908 (29.25)	−51.223 to 61.940	0.083 (0.097), −0.106 to 0.273
Proportion of hatched eggs	0.714 (0.311), n = 55	0.588 (0.364), n = 64	0.166 (0.619)	−0.061 to 0.345	0.367 (0.185), 0.004 to 0.731
Proportion of fledglings	0.518 (0.385), n = 61	0.363 (0.377), n = 69	0.988 (0.627)	−0.251 to 2.254	0.404 (0.177), 0.056 to 0.752

Descriptive statistics are reported as means (SD) and sample sizes for each type of mating system. Effect sizes (β) and associated 95% credible intervals (CI) are shown for the difference between types of social mating system in relation to the breeding parameters. We present Cohen's d effect sizes (with associated standard error (SE) and 95% confidence intervals (95% CI)) for the difference between polyandrous and monogamous nests calculated from the raw data.

### Social mating systems and breeding parameters

**Figure 3:**
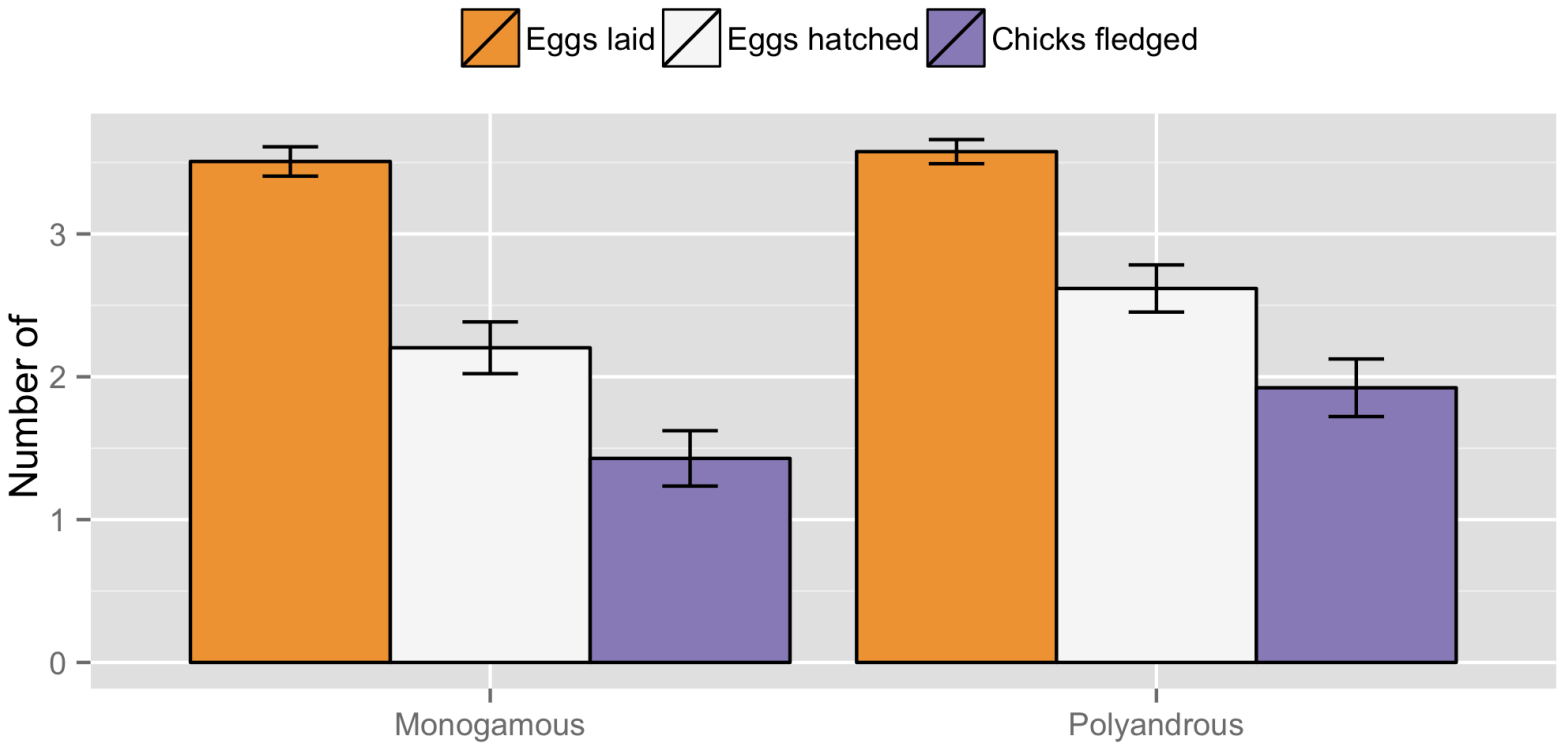
Estimates of breeding parameters by mating system in dunnocks. The mean number of eggs laid, hatched, and chicks fledged per nest by type of social mating system at 158 active dunnock nests in Dunedin, New Zealand (2009–2012). The error bars represent standard errors.

Eggs that successfully hatched were laid over an average period of 2.87 (1.254) days (one egg was laid daily until clutch completion), incubated on average for 12.62 (1.624) days, and chicks fledged on average 12.17 (1.617) days after hatching. Completed clutches ranged in size from one to five eggs. The effect sizes of mating system on laying date of the first egg, clutch size, and egg volume were ‘small’ (all *d*<0.3; Table 1; Figure 3), and the differences between polyandrous and monogamous groups were statistically non-significant. The estimate of *d* was positive for the difference in the proportion of eggs that hatched between polyandrous and monogamous nests, indicating that polyandrous nests hatched a greater proportion of eggs per clutch than monogamous nests (*d* = 0.367; Table 1; Figure 3). This *d* estimate can be considered ‘small’ to ‘medium’ [35], which suggests that the effect of polyandry on hatching success is weak. Among the known reasons associated with eggs failing to hatch were: nest desertion, depredation, infertility, and embryo mortality (Table 2). The probability that eggs failed to hatch due to infertility presented a negative, ‘medium’ to ‘large’, *d* estimate, indicating that it was higher in monogamous nests than in polyandrous ones (*d* = −0.642; Table 2) although the difference was not statistically significant. All other causes showed ‘small’ to ‘medium’ differences and were not significantly different between the two mating systems (Table 2).

**Table 2:** Causes of egg hatching failure by the type of social mating system in dunnocks.

	Polyandrous (%)	Monogamous (%)	Effect size (β (SD))	95% CI	Cohen's d (SE), 95% CI
Desertion	10 (18.18)	11 (12.64)	0.378 (0.515)	−0.542 to 1.406	0.307 (0.439), −0.554 to 1.169
Depredation	14 (25.45)	13 (14.94)	0.701 (0.498)	−0.261 to 1.660	0.525 (0.392), −0.243 to 1.294
Infertility	12 (21.81)	32 (36.78)	−0.858 (0.444)	−1.719 to 0.033	−0.642 (0.345), −1.319 to 0.035
Embryo mortality	8 (14.54)	14 (16.09)	−0.180 (0.530)	−1.239 to 0.849	−0.144 (0.443), −1.014 to 0.725
Unknown	11 (20.0)	17 (19.54)	−0.029 (0.489)	−0.957 to 0.977	−0.022 (0.386), −0.780 to 0.736

The number of eggs and percentages are shown for each category of hatching failure for polyandrous and monogamous nests. Effect sizes (β) and associated 95% credible intervals (CI) are shown for the difference between types of social mating system in relation to the kind of hatching failure. We present Cohen's d effect sizes (with associated standard error (SE) and 95% confidence intervals (95% CI)) for the difference between polyandrous and monogamous nests calculated from the *z*-value of the models and sample sizes from raw data.

Overall, 50% of nests (81/162) successfully fledged at least one offspring. The *d* estimate was positive for the difference in the proportion of chicks that fledged in polyandrous nests in relation to monogamous nests (*d* = 0.404; Table 1; Figure 3). This *d* estimate is considered ‘medium’ [35], which suggests that the effect of the social mating system on fledging success is moderate. The composite *d* estimate of hatching and fledging success was considered ‘medium’ and its 95% CI did not overlap zero (*d* = 0.385, 95% CI: 0.307 to 0.463), which indicates that overall socially polyandry has a moderate effect on the reproductive success.

Of the nests that failed to fledge at least one chick, 28 failed to hatch any eggs, 10 were abandoned, and 20 were predated. We estimated the DSR (daily survival rate) of dunnock nests as a function of the type of social mating system. Nests attended by socially polyandrous pairs had higher DSR than those attended by socially monogamous groups in the first two seasons, but in season C the DSR of monogamous nests was higher than that of polyandrous (Season A: DSR polyandrous  = 0.992 (0.005); DSR monogamous  = 0.982 (0.006); Season B: DSR polyandrous  = 0.989 (0.003); DSR monogamous  = 0.973 (0.006); and Season C: DSR polyandrous  = 0.971 (0.007); DSR monogamous  = 0.979 (0.002)).

## Discussion

In this study, we described the previously unavailable breeding ecology of dunnocks in New Zealand. More importantly, we investigated whether the types of social mating system affected breeding parameters and nest survival in our population. Our results indicate that social polyandry is beneficial for females given that polyandrous nests hatch more eggs and fledge more nestlings than socially monogamous nests. In the following paragraphs, we will discuss these results and their implications.

We observed socially polygynandrous, monogamous and polyandrous mating systems, but did not observe any cases of polygyny. The fact that we only observed these three types of mating system differs from European studies of dunnocks, especially the well-studied population in Cambridge (see [2] for a summary of this study), where all types of social mating systems have been observed. Nevertheless, it is important to note that even in the studies that observed polygynous breeding groups [10], [12], [18], polygyny accounted for only 4 to 8% of the observed breeding systems. More recently, polygynous breeding groups were observed in our New Zealand population (Holtmann, pers. comm.). During this breeding season, polygynous groups accounted for 3% of the breeding groups, and the adult sex ratio was of 0.569 (Holtmann, pers. comm.). All these populations have male-biased adult sex ratios that range from 0.545 to 0.583 (males/all adults), suggesting that males can only achieve polygyny, the breeding system that potentially yields the highest fitness payoffs to males, under very particular scenarios. It seems plausible that the adult sex ratio could potentially explain the difference in the frequency and types of mating systems between dunnock populations. It has been recently shown, across bird species, that adult sex ratio is associated with social mating systems, in such a way that when the sex ratio is male biased, female polygamy is more frequent [37]. Given their variable mating system, dunnocks provide a rare opportunity to test this prediction intra-specifically. A direct consequence of the variable mating system in male-biased populations, is the occurrence of polyandrous breeding systems in which females can count with paternal care provided by two or more males.

It has been previously shown that female dunnocks breeding in polyandrous groups have higher productivity than monogamous females [9]. We found little evidence for differences in clutch size, and egg volume between polyandrous or monogamous nests. These results indicate that polyandrous and monogamous female dunnocks have similar initial investment in the egg stage, and are not allocating resources differentially to eggs according to expectations of future help (see [38], [39] for discussions of load lightening and differential allocation). Nevertheless, polyandrous groups hatched and fledged more chicks than monogamous pairs, as suggested by the moderate positive composite *d* estimate, demonstrating that having more adults attending the nest is beneficial. The benefits of having more males in a breeding groups in dunnocks is similar to the effect of having more helpers-at-the-nest in classic cooperative breeding systems (see [40]–[44]). This finding was supported by the higher daily survival rate of polyandrous nests than that of monogamous ones. This evidence further demonstrates (see [2]) that in the dunnock, breeding in a polyandrous social group provides direct benefits to breeding females. It will be interesting in the future to investigate whether indirect (genetic) benefits also occur as a consequence of breeding polyandrously [5], or whether female age can affect the mating system in which she breeds or how much investment she provides at the egg stage.

While in the dunnock, two studies have found evidence of direct fitness benefits of social polyandry (current study and [9]), studies of other bird species with variable mating system have failed to do so. Both the ground tit [7], and the moustached warbler [8] exhibit variable mating systems, with some females breeding with more than one partner. However in these latter species, the formation of the polyandrous group differs from that of the dunnock. While in dunnocks, the polyandrous males generally form the bond with the female at the beginning of the breeding season, in ground tits and moustached warblers, polyandrous group formation occurs after the female has initiated incubation [7], [8]. It seems reasonable that differences in adult sex ratio among these species (see discussion above) create scenarios in which males: (i) are constrained into forming breeding coalitions (e.g., as in dunnocks) and thus contribute more to paternal care; and (ii) have opportunities to breed monogamously (e.g., as in ground tits and moustached warblers), but when faced with perturbations, such as nest failures, can join another pair and try to secure residual reproductive success. Under scenario (ii), joining males would not be compelled to supply high levels of paternal care, given the low certainty of paternity from joining the pair at such late stage (for a discussion of when males should reduce paternal care, see [45]).

The results of this study allow us to describe, for the first time, important reproductive aspects of the dunnock, one of the most common introduced species of birds in New Zealand. The dunnock has been and is the focus of extensive behavioural ecology studies that investigate aspects of parental care and sexual conflict (e.g., current study, and see [46], [47]). The data presented here represent the first part of results from on-going studies of the genetic mating system, parental investment, and genetic and morphological divergence between NZ and UK dunnocks. These data will provide fruitful contrasts between dunnocks in their native environment with a population introduced to a new environment, providing a rare replication of behavioural ecological studies. Finally, the variable mating system of dunnocks provides opportunities for females to breed polyandrously, which lead to direct fitness benefits.
